# Rural and urban differences in treatment status among children with surgical conditions in Uganda

**DOI:** 10.1371/journal.pone.0205132

**Published:** 2018-11-01

**Authors:** Ashley Bearden, Anthony T. Fuller, Elissa K. Butler, Tu Tran, Fredrick Makumbi, Samuel Luboga, Christine Muhumuza, Vincent Ssennono, Moses Galukande, Michael Haglund, Emily R. Smith

**Affiliations:** 1 Robbins College of Health and Human Sciences, Baylor University, Waco, United States of America; 2 Division of Global Neurosurgery and Neurology, Duke University, Durham, NC, United States of America; 3 Duke University Global Health Institute, Durham, NC, United States of America; 4 University of Minnesota Medical School, Minneapolis, Minnesota, United States of America; 5 Department of Surgery, University of Washington, Seattle, WA, United States of America; 6 Makerere University School of Public Health, Kampala, Uganda; 7 Department of Anatomy, Makerere University School of Medicine, Kampala, Uganda; 8 Ministry of Health, Government of Uganda, Kampala, Uganda; 9 Department of Surgery, Makerere University College of Health Sciences, Kampala, Uganda; 10 Department of Neurosurgery, Duke University Medical Center, Durham, NC, United States of America; Indiana University, UNITED STATES

## Abstract

**Background:**

In low and middle-income countries, approximately 85% of children have a surgically treatable condition before the age of 15. Within these countries, the burden of pediatric surgical conditions falls heaviest on those in rural areas. The objective of the current study was to evaluate the relationship between rurality, surgical condition and treatment status among a cohort of Ugandan children.

**Methods:**

We identified 2176 children from 2315 households throughout Uganda using the Surgeons OverSeas Assessment of Surgical Need (SOSAS) survey. Children were randomly selected and were included in the study if they were 18 years of age or younger and had a surgical condition. Location of residence, surgical condition, and treatment status was compared among children.

**Results:**

Of the 305 children identified with surgical conditions, 81.9% lived in rural areas. The most prevalent causes of surgical conditions reported among rural and urban children were masses (24.0% and 25.5%, respectively), followed by wounds due to injury (19.6% and 16.4%, respectively). Among children with untreated surgical conditions, 79.1% reside in rural areas while 20.9% reside in urban areas. Among children with untreated surgical conditions, the leading reason for not seeking surgical care among children living in both rural and urban areas was a lack of money (40.6% and 31.4%, respectively), and the leading reason for not receiving care in both rural and urban settings was a lack of money (48.0% and 42.8%, respectively).

**Conclusions:**

Our data suggest that over half of the children with a surgical condition surveyed are not receiving surgical care and a large majority of children with surgical needs were living in rural areas. Future interventions aimed at increasing surgical access in rural areas in low-income countries are needed.

## Introduction

Approximately 5 billion do not have timely, safe, and affordable access to surgical care, with the highest estimated need in sub-Saharan Africa. [1, 2] The absence of surgical care in these regions lends to higher rates of morbidity and mortality as well as increased disability adjusted life years (DALYs).[3] By incorporating essential surgical care into low-income and middle-income countries (LMICs) health strategic plans, approximately 1.5 million deaths a year could be avoided, which equates to 6–7% of all avertable deaths in LMICs.[4] In addition access to adequate essential surgical care is vital to achieve the Sustainable Development Goals (SDGs), as advocated for by the World Health Organization, the World Bank, and the United Nations.[5–7]

The surgical burden among children is great with approximately 10-85% of children in LMICs having a surgically treatable condition before the age of 15.[8] According to one study spanning across four LMICs (Rwanda, Uganda, Sierra Leone, and Nepal), the highest unmet surgical need among the pediatric population was due to head, face and neck conditions and one-third of all conditions were due to masses.[9] In addition, an estimated 303,000 children die within four weeks of birth due to congenital anomalies and many of these structural congenital anomalies could be treated with surgical care.[10] However throughout Sub-Saharan Africa, many children lack access to surgical care, leading to increased rates of life-long disability and mortality.[11] Within Uganda, the site of the current study, approximately 1.3 million children currently have an untreated surgical condition, with the highest need in the rural Northern and Western regions.[12, 13]

Within LMICs, treatment status by geographic location is an important factor that needs to be taken into consideration regarding the high unmet pediatric surgical need.[14] The receipt of appropriate surgical treatment among children within LMICs are not evenly distributed within urban and rural communities. The Lancet Commission on Global Surgery advocates for a 2-hour travel distance for 80% of the population to a surgical facility.[3] However, many communities in rural areas live well beyond the 2-hour travel distance recommendation and can live days away from surgical centers. [3] Furthermore, the median distance to a hospital for LMICs ranges from approximately 10km to 70km, with longer distances associated with rural communities.[3]

The objective of the current study was to evaluate the relationship between rurality, surgical condition and treatment status among a cohort of Ugandan children, using a population-based, community survey.

## Methods

### Setting

Uganda is a low-income country with a population of approximately 39 million people and roughly 83% of the population is living in rural areas.[15, 16] Over 55% of the population is under the age of 18 and the country has one of the highest birth rates.[17] Uganda is divided into four demographic regions: central, eastern, northern, and western, and the capital city of Kampala, located in the central region, has the highest urban population density with approximately 1.35 million or 3.1% of the population residing.[17] The health system of Uganda includes a private and a public sector. The public sector is comprised of different levels: national referral hospitals, regional referral hospitals, and district hospitals and health centers.[18] The majority of care in Uganda is provided at the district hospital level and there are approximately 0.1 surgeons per every 100,000 people.[19]

### Data collection

A nationwide, two-stage cluster-randomized household survey was administered in 105 enumeration areas, clustered by geographic sub-region, in Uganda using the Surgeons OverSeas Assessment of Surgical Need (SOSAS) survey, described in detail elsewhere.[15] In short, 105 enumeration areas were selected using probability of selection proportional to population size within each region. Household selections within each enumeration area was done by simple random sampling from complete household listings. The SOSAS survey is a validated questionnaire used to identify the prevalence of surgical conditions in a region. The SOSAS instrument is given in two portions. The first portion of the SOSAS instrument is a survey given to a household representative to establish household demographics, deaths within the past year, and what conditions accompanied those deaths. The second section consists of two randomly selected household members undergoing an interview inquiring about surgical conditions in the body. Data were collected by means of trained enumerators living in each enumeration area.

### Ethical considerations

The Makerere University School of Medicine Research and Ethics Committee, Duke University Health System Institutional Review Board and University of Minnesota Institutional Review Board approved this study prior to implementation. Enumerators obtained informed consent from each head of household and each individual participating in the survey, prior to the initiation of the survey. For children less than the age of 18, a parent or guardian provided informed consent, and children ages 8 to18 provided assent. When necessary, parents or guardians assisted children in answering survey questions. If an identified surgical condition was identified in the home, a referral to a surgeon at the national hospital was provided. In addition, information on the nearest health facility that had surgical management was also provided.

### Statistical analysis

Children were defined as respondents in the study between the ages of 0 and 18 years. Surgical conditions were self-reported by the survey respondents. A group of surgical trainees and surgeons assessed each recorded surgical condition and assigned a rating on a four-point scale as to whether to condition was definitely not surgical, likely not surgical, likely surgical, and definitely surgical. For discordant coding, the case was discussed and given a final score based on consensus. Cases with a score of likely surgical or definitely surgical were defined as surgically treatable conditions. Each case was also coded to indicate whether the patient receive surgical care through a minor or major operation.

Quantitative analyses were conducted using SAS v9.4 (SAS Institute Inc., Cary, NC, USA), and Data were stored in spreadsheets on Microsoft Excel 2010 (Microsoft Corp, Redmond, WA, USA). Statistical significance was defined at the two-sided α = 0.05 level. Using design weights, subjects at the household and individual-level were weighted for each enumeration area by known population counts for gender and age groupings in the Uganda Census 2014 data. andLocation of residence, surgical condition, and treatment status was compared among children.

Univariate models of demographic characteristics, rural and urban status and treatment status were calculated using chi-square tests for categorical data and t-tests for continuous data. Demographic Data were compared between children that reported surgical conditions that were treated or untreated and between children whose location of residence was either rural or urban. Barriers to surgery were calculated using chi-square tests, by stratifying barriers into urban and rural status, as well as seeking and receiving surgical care. If respondents had multiple responses and barriers each barrier was counted and analyzed in its individual category and not as a multifaceted answer.

## Results

The SOSAS survey identified 2,176 children among 4,428 respondents (49.1%) from 2,315 households across 105 enumeration areas throughout Uganda. From the 2,176 children identified through the survey, 305 children reported having a surgical condition. Of the 305 children, 250 children (81.9%) reside in rural areas, while 55 (18.1%) reside in urban locations (Table 1). The rural/urban distribution in the study sample of 305 were representative of the overall sample of 2,176 children in the original SOSAS survey. Of the 2,176 children, 81.8% lived in rural areas and 18.2% lived in urban areasThe most prevalent age group of those with surgical conditions interviewed were those in the 0-5 year age group, followed by those in the 10-14 year age group (35.0% and 29.5%, respectively). The type of condition present was significant between the rural and urban areas (p = .05), with the most prevalent rural and urban conditions being masses (24.0% and 25.5%, respectively). For rural dwellers, the face (16.8%) was the second most prevalent area with a reported surgical condition, while the abdomen (18.2%) was the second most prevalent area with a surgical condition among urban respondents. The majority (52.4%) of pediatric patients with a surgical condition reported the condition currently being present. Of the rural respondents, 49.6% reported the condition as currently present and 61.8% of urban respondents reported a currently present condition (p = .08). Approximately half of rural children (58.0%) received either a minor or major surgical procedure (52.0% and 6.0%, respectively), and approximately half of urban children (54.5%) received a minor or a major surgical procedure (50.0% and 3.6%, respectively).

**Table 1:** Demographic characteristics of children with surgical needs, by location of residence.

	Total	Rural	Urban	P value*
	(n = 305)	(n = 250)	(n = 55)	
*Demographic characteristics*	n (%)	n (%)	n (%)	
Age (years)				0.59
0-5	95 (35.0)	82 (32.8)	13 (23.6)	
6-9	56 (17.1)	44 (17.6)	12 (21.8)	
10-14	86 (29.5)	69 (27.6)	17 (30.9)	
15-18	68 (18.3)	55 (22.0)	13 (23.6)	
Gender				0.82
Male	151 (51.3)	123 (49.2)	28 (50.9)	
Female	154 (48.7)	127 (50.8)	27 (49.1)	
Healthy in past 12 months				0.53
Yes	232 (74.7)	192 (76.8)	40 (72.7)	
No	73 (25.3)	58 (23.2)	15 (27.3)	
Facility visits in past 12 months				0.43
0	250 (81.2)	208 (83.2)	42 (76.4)	
1-3	41 (14.2)	32 (12.8)	9 (16.4)	
>4	14 (4.6)	10 (4.0)	4 (7.2)	
*Condition characteristics*				
Anatomical region				0.55
Extremities	90 (31.6)	78 (31.2)	12 (21.8)	
Face	50 (14.5)	42 (16.8)	8 (14.5)	
Head	45 (13.9)	38 (15.2)	7 (12.7)	
Buttocks	35 (12.4)	27 (10.8)	8 (14.5)	
Abdomen	30 (10.1)	20 (8.0)	10 (18.2)	
Chest	16 (5.7)	12 (4.8)	4 (7.3)	
Neck	16 (5.0)	14 (5.6)	2 (3.6)	
Groin (Male)	11 (3.5)	9 (3.6)	2 (3.6)	
Back	6 (1.6)	5 (2.0)	1 (1.8)	
Breast	3 (0.9)	3 (1.2)	0	
Groin (Female)	3 (0.8)	2 (0.8)	1 (1.8)	
Present now (yes)				0.08
Yes	158 (52.4)	124 (49.6)	34 (61.8)	
No	147 (47.6)	126 (50.4)	21 (38.2)	
Timing of onset				0.19
<1 month	46 (15.9)	41 (16.4)	5 (9.1)	
1-12 months	77 (23.8)	65 (26.0)	12 (21.8)	
>12 months	182 (60.3)	144 (57.6)	38 (69.1)	
Type of condition				0.05
Masses	74 (25.6)	60 (24.0)	14 (25.5)	
Wound (injury)	58 (18.6)	49 (19.6)	9 (16.4)	
Acquired deformity	52 (15.9)	47 (18.8)	5 (9.1)	
Wound (not injury)	46 (14.4)	38 (15.2)	8 (14.5)	
Burn	32 (11.2)	24 (9.6)	8 (14.5)	
Congenital deformity	23 (8.3)	19 (7.6)	4 (7.3)	
Abdominal problems	18 (5.6)	13 (5.2)	5 (9.1)	
Genitalia/urinary	2 (0.4)	0 (0.0)	2 (3.6)	
*Health care sought / care received*				
Health care sought				0.32
Yes	223 (71.5)	186 (74.4)	37 (67.3)	
No	82 (28.5)	64 (25.6)	18 (32.7)	
Traditional healer sought				0.55
Yes	62 (21.2)	50 (20.0)	12 (21.8)	
No	240 (78.8)	197 (78.8)	43 (78.2)	
Type of care received				0.76
No care	119 (31.8)	97 (38.8)	22 (40.0)	
Minor procedure	158 (51.1)	130 (52.0)	28 (50.9)	
Major procedure	17 (5.1)	15 (6.0)	2 (3.6)	
Referred	11 (4.0)	8 (3.2)	3 (5.5)	

*P-value assessing difference between rural and urban children with a surgical condition.

Among children residing in rural areas who reported a surgical condition (n = 250), 126 (50.4%) of reported having a surgical procedure and 124 (49.6%) reported not having a surgical procedure (Table 2). Of the children in urban locations who reported a surgical condition (n = 55), 23 (41.8%) reported having a surgical procedure while 32 (58.2%) reported not having a surgical procedure. The most prevalent anatomical region to have an untreated surgical condition in rural areas was the extremities (32.7%), while it was the face (23.3%) in urban children (Table 2). For treated conditions, the most common anatomical region for a surgical condition was the extremities for both rural and urban children (33.6% and 34.2%, respectively). Among children residing in rural areas, children with untreated surgical conditions reported that the surgical condition was currently present (75.8%, p <.0001), while in children residing in urban areas, children with an untreated surgical condition reported that the surgical condition was currently present (90.9%, p <.0001). The most prevalent causes of surgical conditions reported among rural and urban children were masses (24.0% and 25.5%, respectively), followed by wounds due to injury (19.6% and 16.4%, respectively).

**Table 2:** Demographic characteristics and condition characteristics of children interviewed in SOSAS, stratified by urban/rural village type and treatment status.

	Rural				Urban			
	Total	Treated surgical conditions	Untreated surgical conditions	P value*	Total	Treated surgical conditions	Untreated surgical conditions	P value*
	(n = 250)	(n = 126)	(n = 124)		(n = 55)	(n = 23)	(n = 32)	
*Demographic characteristics*	n (%)	n (%)	n (%)			n (%)	n (%)	
Age (years)								
0-5	82 (32.8)	38 (32.7)	44 (39.4)	0.32	13 (23.6)	5 (27.6)	8 (32.5)	0.79
6-9	44 (17.6)	22 (16.2)	22 (17.1)		12 (21.8)	4 (16.2)	8 (21.6)	
10-14	69 (27.6)	34 (28.7)	35 (29.3)		17 (30.9)	7 (32.0)	10 (31.5)	
15-18	55 (22.0)	32 (22.5)	23 (14.1)		13 (23.6)	7 (24.1)	6 (14.4)	
Gender								
Male	123 (49.2)	62 (51.0)	61 (50.7)	0.97	28 (50.9)	13 (61.1)	15 (48.0)	0.31
Female	127 (50.8)	64 (49.0)	63 (49.3)		27 (49.1)	10 (38.9)	17 (52.0)	
Healthy in past 12 months								
Yes	192 (76.8)	111 (87.7)	81 (62.3)	0.0006	40 (72.7)	19 (82.9)	21 (66.0)	0.37
No	58 (23.2)	15 (12.3)	43 (37.7)		15 (27.3)	4 (17.1)	11 (34.0)	
Facility visits in past 12 months								
0	208 (83.2)	113 (88.8)	95 (75.4)	0.05	42 (76.4)	19 (82.9)	23 (72.9)	-
1-3	32 (12.8)	10 (9.0)	22 (18.0)		9 (16.4)	4 (17.1)	5 (17.8)	
>4	10 (4.0)	3 (2.2)	7 (6.7)		4 (7.2)	0 (0.0)	4 (9.3)	
*Condition characteristics*								
Anatomical region				-				-
Extremities	78 (31.2)	38 (33.6)	40 (32.7)		12 (21.8)	6 (34.2)	6 (18.4)	
Face	42 (16.8)	24 (16.4)	18 (12.7)		8 (14.5)	0 (0.0)	8 (23.3)	
Head	38 (15.2)	22 (17.3)	16 (11.1)		7 (12.7)	5 (19.6)	2 (8.4)	
Buttocks	27 (10.8)	18 (15.5)	9 (9.1)		8 (14.5)	5 (20.1)	3 (7.9)	
Abdomen	20 (8.0)	4 (2.7)	16 (14.4)		10 (18.2)	4 (14.7)	6 (19.5)	
Neck	14 (5.6)	8 (5.7)	6 (4.6)		2 (3.6)	0 (0.0)	2 (6.9)	
Chest	12 (4.8)	1 (0.8)	11 (9.6)		4 (7.3)	2 (9.0)	2 (7.6)	
Groin (Male)	9 (3.6)	3 (2.7)	6 (4.4)		2 (3.6)	0 (0.0)	2 (5.5)	
Back	5 (2.0)	5 (3.4)	0 (0.0)		1 (1.8)	1 (2.3)	0 (0.0)	
Breast	3 (1.2)	2 (1.6)	1 (0.5)		0 (0.0)	0 (0.0)	0 (0.0)	
Groin (Female)	2 (0.8)	1 (0.4)	1 (1.0)		1 (1.8)	0 (0.0)	1 (2.5)	
Present now (yes)				<.0001				<.0001
Yes	124 (49.6)	32 (25.4)	92 (75.8)		34 (61.8)	5 (17.9)	29 (90.9)	
No	126 (50.4)	94 (74.6)	32 (24.2)		21 (38.2)	18 (82.1)	3 (9.1)	
Timing of onset								
<1 month	41 (16.4)	21 (18.8)	20 (16.0)		5 (9.1)	0 (0.0)	5 (15.2)	
1-12 months	65 (26.0)	34 (25.7)	31 (23.7)		12 (21.8)	9 (37.5)	3 (7.9)	
>12 months	144 (57.6)	71 (55.5)	73 (60.3)		38 (69.1)	14 (62.5)	24 (76.9)	
Type of condition				-				-
Masses	60 (24.0)	26 (22.0)	34 (27.5)		14 (25.5)	4 (23.1)	10 (34.2)	
Wound (injury)	49 (19.6)	37 (29.3)	12 (9.2)		9 (16.4)	5 (20.7)	4 (12.0)	
Acquired deformity	47 (18.8)	20 (15.1)	27 (20.8)		5 (9.1)	1 (2.3)	4 (10.0)	
Wound (not injury)	38 (15.2)	24 (17.9)	14 (10.9)		8 (14.5)	5 (20.7)	3 (9.7)	
Burn	24 (9.6)	14 (12.3)	10 (8.3)		8 (14.5)	6 (28.8)	2 (6.0)	
Congenital deformity	19 (7.6)	2 (1.4)	17 (14.7)		4 (7.3)	0 (0.0)	4 (15.6)	
Abdominal problems	13 (5.2)	3 (1.9)	10 (8.7)		5 (9.1)	1 (2.2)	4 (10.1)	
Genitalia/urinary	0 (0.0)	0 (0.0)	0 (0.0)		2 (3.6)	1 (2.2)	1 (2.5)	
*Health care sought / care received*								
Health care sought				<.0001				0.0003
Yes	186 (74.4)	116 (92.5)	70 (52.7)		37 (67.3)	22 (91.6)	15 (48.3)	
No	64 (25.6)	10 (7.5)	54 (47.3)		18 (32.7)	1 (8.4)	17 (51.7)	
Traditional healer sought				0.0069				0.66
Yes	50 (20.0)	18 (12.9)	32 (28.5)		12 (21.8)	4 (26.2)	8 (21.8)	
No	197 (78.8)	107 (87.1)	90 (71.5)		43 (78.2)	19 (73.8)	24 (78.2)	
Type of care received				-				-
No care	97 (38.8)	11 (8.3)	86 (70.5)		22 (40.0)	2 (14.0)	20 (62.2)	
Minor procedure	130 (52.0)	103 (82.2)	27 (21.4)		28 (50.9)	20 (83.8)	8 (21.6)	
Major procedure	15 (6.0)	12 (9.5)	3 (1.9)		2 (3.6)	1 (2.2)	1 (2.3)	
Referred	8 (3.2)	0 (0.0)	8 (6.2)		3 (5.5)	0 (0.0)	3 (13.9)	

*P-value assessing difference between untreated and treated conditions, by rural or urban residence.

**Fig 1:**
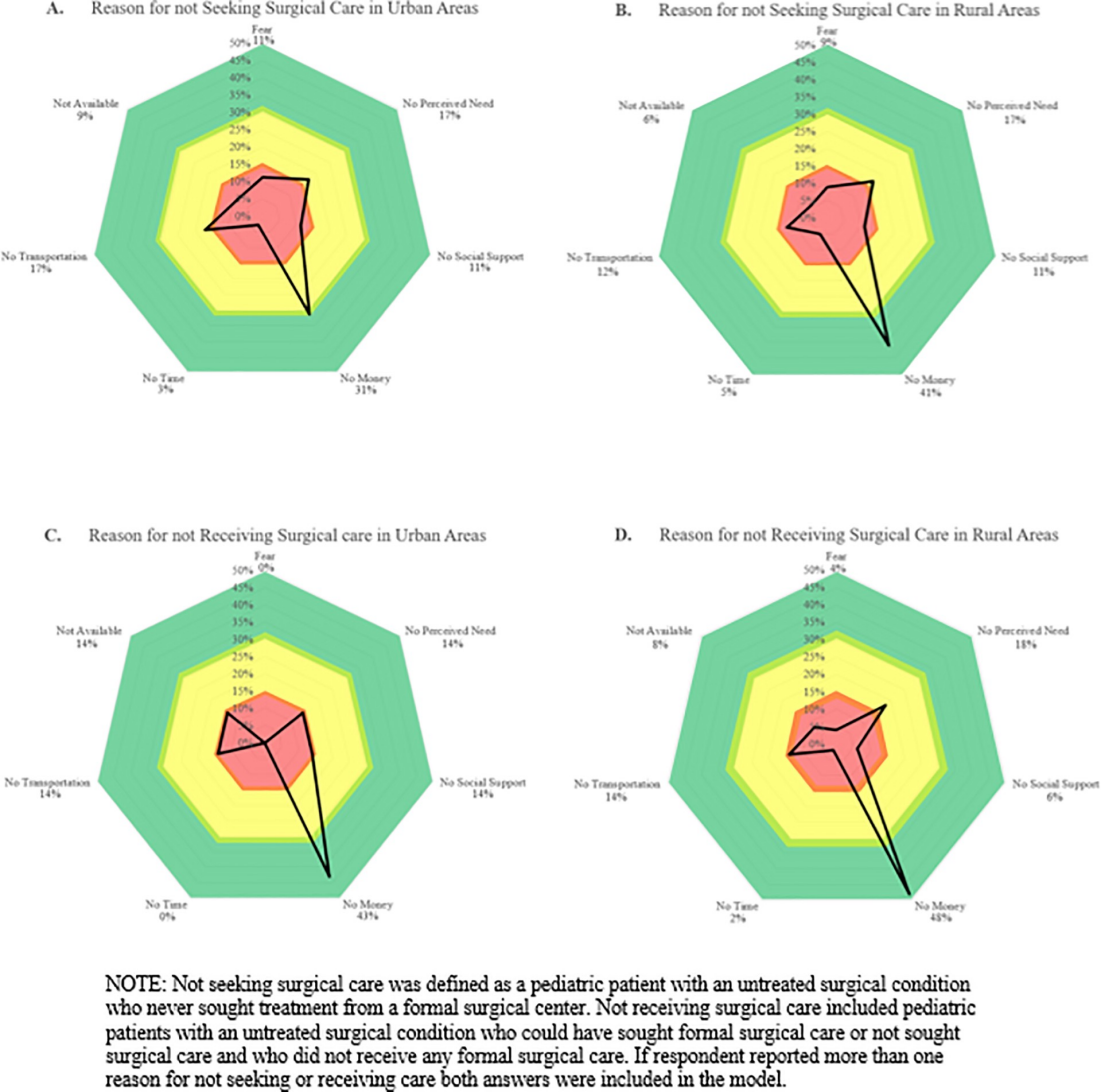
Reasons for not seeking surgical care and reasons for not receiving surgical care in a cohort of Ugandan children with identified surgical needs (n = 305).

Among children with untreated surgical conditions, the leading reason for not seeking surgical care among children living in both rural and urban areas was a lack of money (40.6% and 31.4%, respectively) (Fig 1). In urban areas, a lack of transportation and no perceived need (17.1% and 17.1%, respectively) were the second most common response for not seeking surgical care. In rural areas, the second most common response for not seeking surgical care was no perceived need (16.5%). Among children with untreated surgical conditions the leading reason for not receiving care in both rural and urban settings was a lack of money (48.0% and 42.8%, respectively). Urban areas also reported that a lack of social support (14.3%), no perceived need (14.3%), a lack of transportation (14.3%), and surgical care not being available (14.3%) as reasons for not receiving surgical care. Urban residents, however, did not report fear or time as a reason for not receiving surgical care. In rural areas, no perceived need (18.0%), no transportation (14.0%), surgical care not being available (8.0%), no social support (6.0%), fear (4.0%), and no time (2.0%) were all reported as reasons for not receiving surgical care (Fig 1).

## Discussion

Within LMICs the burden that is placed on families, children, and the healthcare system is far outweighing the current capacity for treatment. With a limited surgical workforce, the unmet need in Uganda is continuing to grow and the surgical need will continue to be high.[11, 20] Our study shows that among children with a surgical condition in Uganda, over 80% live in rural areas. By extrapolating this estimate to the current population using the 2015 Uganda census data [21] and of those that currently have a surgical condition, we find that just over 1 million children living in rural areas have a current surgical need and a little over half are untreated. Our data also suggests that there needs to be an improved access to healthcare and surgical services in all areas of the country.

While approximately 75% of the children with surgical needs in our survey sought formal healthcare, nearly half didn’t receive care even when it was sought out, regardless if they lived in a rural or urban area. This addresses that there is a need for care at the lower levels of the healthcare system in Uganda and highlights the need for health system strengthening in LMICs. Within our study, it was found that the largest barrier in both rural and urban areas to seeking and receiving care was financial constraints. These financial constraints are a major concern in pediatric global surgery because many people do not pursue care due to the associated costs.[3] Of those who do seek surgical care, 33 million people globally experience catastrophic expenditure due to surgical costs.[3] Another 48 million face catastrophic expenditure because of other costs associated with receiving care.[3] Catastrophic expenditures for families include the costs of travel, lodging and care. Catastrophic expenditures in relation to surgical care is a topic that is becoming more widely discussed, however little is still being done about this issue.[22, 23] Due to the first two, travel and lodging, it is understandable why those in rural communities are not seeking care when just the simple act of getting there can place them into extreme poverty. This lack of access and funds is highlighted by the lack of resources available for pediatric surgical care in Uganda.[24]

To help resolve this disproportionate relationship when it comes to pediatric surgical care in Uganda and other LMICs, multiple steps and changes need to be made. First, to address the catastrophic expenditures that families are facing, more research needs to be completed around costs of services at hospitals and healthcare locations at all levels of the tiered system.[3] By figuring out how prices can be reduced for families both medically and non-medically, health seeking behavior may improve and some of the risk of catastrophic expenditure would be reduced.[25] Although all services at public facilities in Uganda are provided nominally free of charge, families are typically asked to purchase consumables, such as sutures, implantable mesh, and anesthetic medications, creating significant financial barriers. A second area that should be focused on, is increasing the number of surgeons and increasing the size of the surgical workforce that are able to perform surgical procedures.[26–28] Within LMICs the burden that is placed on families, children, and the healthcare system is far outweighing the current capacity for treatment. With a limited surgical workforce, the unmet need in Uganda is continuing to grow and the surgical need will continue to be high.[11, 20] Not only is there a disproportionate surgical need among children in Uganda, that need is even greater among those living in rural areas, especially since most of the pediatric surgeons are all located in urban capital city of Kampala. Currently, there are only three practicing pediatric surgeons in Uganda and 2 are located in the urban capital city of Kampala.[12] This disparity is similar to other LMICs with a disproportionate number of pediatric surgeons to the children population.[29] While it is helpful having outside help, local surgeons and doctors are always sought after because they understand the communities and customs, so the retention of local surgeons is key.[30] Increasing funding for global health research and global pediatric surgery is another necessity. By increasing the funds more research and implementation can be put into place.[31, 32] Overall, access and cost of care need to be addressed to help end this disproportionate issue with pediatric surgery.

The primary limitations to this study are those inherent to cross-sectional surveys. Due to the nature of self-reporting individuals may overlook certain conditions and leave them out due to not perceiving them as surgical or not knowing of a condition. However, in a recent study using SOSAS, approximately 95% of cases were reliable using both oral self-reporting and visual physical examinations by physicians.[33] The second limitation was that primary data collectors were not medically trained physicians. These limitations were addressed by receiving a full detailed verbal description of the condition reported and then having medical staff evaluate which conditions were surgical based off reported symptoms. A limitation inherent to our sampling methodology was selecting the districts proportional to population. Thus, districts with larger cities were more likely to be selected for inclusion. Our results may be an underestimate of the actual burden in rural areas who did not receive surgical care due to accessibility issues, which is often a barrier in rural areas to receiving surgical care. However, in the parent SOSAS study, rural regions were oversampled to account for key demographic differences. No differences were found between the study population and overall general population in Uganda in terms of median household size, gender proportions, and contraceptive use among women.[34] Although there is potential for selection bias in all household surveys, we assumed the risk of bias is low given no differences were found in key demographic variables.

## Conclusion

According to our results, over half of the children with a surgical condition surveyed are not receiving surgical care and a large majority of these children were living in rural areas. Future interventions aimed at increasing surgical access in rural areas for children are needed. Researchers must also focus on the catastrophic expenditures that families are facing to get care, because according to our data the leading barrier to not receiving or seeking care is cost related.
